# Efficacy of inhaled Desmopressin in pregnant women with idiopathic oligohydramnios – a randomized controlled trial

**DOI:** 10.25122/jml-2021-0141

**Published:** 2022-11

**Authors:** Marziye LotfAlizadeh, Somayeh MoeinDarbari, Neshat MohebbanAzad, Nayereh Ghomian

**Affiliations:** 1 Department of Obstetrics and Gynecology, Faculty of Medicine, Mashhad University of Medical Sciences, Mashhad, Iran

**Keywords:** oligohydramnios, Desmopressin, amniotic fluid

## Abstract

The aim of this study was to investigate the therapeutic effect of inhaled Desmopressin (DDAVP) in pregnant women with idiopathic oligohydramnios. This randomized, double-blind clinical trial involved 44 pregnant women at 28–37 weeks of gestation with idiopathic oligohydramnios admitted in 2 academic hospitals in Mashhad, Iran, from 2018 to 2019. In the intervention group, 10µg DDAVP was nasally sprayed. The control group received intravenous maintenance fluid. The hematocrit, electrolytes, blood pressure and urine-specific gravity were evaluated at baseline and 3, 8, and 24 hours later. Amniotic fluid index (AFI) was measured using ultrasound at baseline, 24 and 48 hours later. There was no significant difference in the basic characteristics (age, body mass index, and gestational age) between the two groups. The pattern of changes of AFI (baseline, 24 and 48 hours later) increased in the intervention (4.16±0.86, 7.08±1.453 and 7.76±1.62, p<0.001) and control groups (4.23±0.70, 5.39±1.079 and 5.68±1.10, p<0.001). Serum sodium levels significantly declined in the intervention group (p<0.001) but not in the control group (p=0.07). There were no significant differences in potassium (p=0.89), hematocrit (p=0.23), systolic blood pressure (p=0.21) and diastolic blood pressure (p=0.97). However, urine-specific gravity had an increasing pattern in the intervention group (p<0.001) and a decreasing pattern in the control group (p<0.001). This study showed that Desmopressin inhalation could increase the AFI and urine specific gravity, enhancing oligohydramnios treatment in pregnant women, compared to serum administration.

## INTRODUCTION

If the volume of amniotic fluid is less than expected at corresponding gestation, the condition is called oligohydramnios. The disorder is easily detected by ultrasound which can qualitatively describe it as normal, decreased or increased [1]. Oligohydramnios is an abnormally decreased amount of amniotic fluid that complicates 1 to 2% of pregnancies. Oligohydramnios sonographic diagnosis is typically based on an amniotic fluid index (AFI) of less than 5 cm or a single deepest pocket of amniotic fluid of less than 2 cm [2]. The assessment of amniotic fluid volume (AFV) is a well-established aspect of the antenatal monitoring of pregnancies at risk for a negative pregnancy outcome. The amniotic fluid index and the single deepest pocket are the most often utilized ultrasonography techniques for estimating AFV (SDP). Four studies have defined normal AFVs, and while their normal volumes are similar, there are discrepancies, owing mostly to the statistical methods utilized in each study [3].

With a 2.3% incidence, oligohydramnios is one of the most common fetal health threats. Oligohydramnios is linked to intrauterine growth limitation, respiratory distress syndrome, post-maturity syndrome, and prolonged fetal hypoxia. Fetal malpresentation, umbilical cord compression, meconium staining, increased perinatal mortality and morbidity, and increased surgical delivery may all be caused by this syndrome [4].

There are various sources of amniotic fluid production, each of which has a diverse role in the different stages of pregnancy. Understanding amniotic fluid production sources help diagnose fluid disturbances [5]. Appropriate fetal growth and development require a normal amniotic fluid volume. The research on amniotic fluid volume regulation could provide crucial insights into how the fetus maintains water homeostasis. Hydrostatic and osmotic forces can influence the process of water flow through a membrane. Changes in the placenta's osmotic/oncotic and hydrostatic forces may affect maternal-fetal water flow. Understanding these pathways may lead to novel treatments for amniotic fluid volume anomalies, resulting in improved clinical outcomes [6].

The process that regulates amniotic fluid volume within acceptable limits is not completely understood. Regulatory mechanisms operate on three levels: placental control of water and solute transport, fetal inflows and outflows, and maternal effect on fetal fluid balance. The chemical composition of its constituents changes with gestational age. Amniotic osmolarity drops slightly when fetal urine enters the amniotic sac compared to fetal blood. With increasing gestational age, amniotic fluid osmolarity reduces further after keratinization of the fetal skin [7]. The concentration of sodium amniotic fluid is much lower than the sodium concentration of embryonic blood. Urine sodium concentration is estimated to be between 20–40% of the amniotic fluid concentration. It should be noted that the sodium concentration of the pulmonary fluid in the fetus is slightly lower than the sodium concentration of the blood. Therefore, the sodium concentration of pulmonary fluid is significantly higher than the amniotic fluid [8]. In addition, pulmonary fluid chloride concentration is approximately twice the concentration in the amniotic fluid chloride. The concentration of urine chloride is very low and is estimated at 10–20% of amniotic fluid chloride concentration [8].

Factors such as fetal abnormalities (congenital abnormalities, chromosomal abnormalities, fetal death, fetal growth restriction, post-term pregnancy, fetal membrane rupture), placental abnormalities (twin-to-twin transfusion, placental abruption,) maternal causes (uteroplacental insufficiency, preeclampsia, hypertension, diabetes), and drug use (inhibitor of prostaglandin synthesis, angiotensin-converting enzyme inhibitor) cause oligohydramnios. Idiopathic oligohydramnios is given when there is no clear cause [9].

In addition, there might be a relationship between oligohydramnios and pregnancy in the summer which can be explained by the water storage of the mother [10]. All pregnant women should be aware of the necessity of adequate hydration. It would, however, be even more important for women carrying pregnancies at risk of uteroplacental insufficiencies because poor fluid intake could lead to additional fetal impairment. Several studies also demonstrated the importance of maintaining appropriate hydration during exercise, temperature exposure, or gastrointestinal loss during pregnancy [11]. Oligohydramnios can also be due to changes in water channels (aquaporin 1 and 2) in the membranes of the embryo and placenta [12].

No effective long-term treatment has been identified for oligohydramnios. However, certain cases can be managed through a temporary increase of amniotic fluid volume accomplished by administering amnioinfusion. In this method, about 200 ml of saline is injected into the amniotic sac by ultrasound guidance. This technique is continuously used to prevent fetal complications in idiopathic oligohydramnios or premature oligohydramnios due to the rupture of membranes [13].

Morad et al. found that nitric oxide donors may be a potential option for enhancing amniotic fluid volume and pregnancy outcomes in cases of isolated oligohydramnios. Nitric oxide causes vasodilation and prevents platelet aggregation. These methods promote uteroplacental perfusion by increasing blood volume and decreasing viscosity in the fetomaternal circulation [14].

In a study by Maher et al., Sildenafil citrate raised amniotic fluid in intricate pregnancies due to oligohydramnios [15]. Phosphodiesterase enzyme inhibitor tablets, now available under the names of Viagra, Tadalafil, Sildenafil etc, are vasodilators. Some new studies have shown that Sildenafil citrate improves vasodilation of tiny myometrial arteries, increasing the amniotic fluid index, fetal weight, and even uterine and umbilical artery Doppler patterns. However, users of Sildenafil may experience headaches, dizziness, flushing, or stomach trouble [16].

Desmopressin (anti-diuretic) and hydration decreased the osmolarity of maternal plasma and fetal plasma in animal studies, and the urinary flow rate of the fetus significantly increased amniotic fluid [15].

Until now, oligohydramnios treatment methods have not been very effective, and it seems essential to look for other successful methods. Since some studies showed that treatment with Desmopressin is not clear, this research aimed to investigate the therapeutic impact of nasal Desmopressin in pregnant females with idiopathic oligohydramnios at 28 to 37 weeks of gestation. The secondary objectives of this work were to define the effect of Desmopressin on blood pressure, hematocrit, plasma sodium and potassium, and urine-specific gravity.

## MATERIAL AND METHODS

### Study design

This randomized, double-blind clinical trial included 44 pregnant women at 28–37 weeks of gestation, diagnosed with idiopathic oligohydramnios, admitted to Imam Reza Hospital and Ummol Banin Hospital of Mashhad, Iran (Southwest Asia) from 2018 to 2019. These are among the seven educational academic hospitals affiliated with Mashhad University of Medical Sciences. Mashhad is the second most populated city (>3 million inhabitants in 2016) in the Islamic Republic of Iran.

### Exclusion and inclusion criteria

The inclusion criteria were pregnancies between 28–37 weeks of gestation with idiopathic oligohydramnios, the viability of the fetus, urine specific gravity below 1010, intact membranes, no intrauterine growth retardation, no anomaly of the fetus, no presence or history of maternal hyponatremia, no reduced moderate to severe renal function (creatinine clearance less than 50 ml/min), and no diagnosed comorbidities like diabetes, preeclampsia, severe anemia, and severe hypertension. The exclusion criteria were delivery date before initiating the treatment with Desmopressin, emergent termination of pregnancy, and hypersensitivity reaction to Desmopressin administration.

### Sample size calculation

Sample size estimation was based on a similar study [17] which reported the changes in the amniotic fluid index (AFI) baseline and after 8h (4.1±0.6 and 8.2±1, respectively). The formula for comparing a quantitative variable in two samples


$$\left(n=\frac{{\left(z_{1\frac{\alpha }{2}}+z_{1-\beta }\right)}^{2}(\sigma_{1}^{2}+\sigma_{2}^{2})}{{\left(\mu_{1}-\mu_{2}\right)}^{2}}\right)$$

was used. Considering α=0.05 and β=0.2, and after considering the dropout rate, 22 patients were evaluated in each group.

### Procedures

240 cases diagnosed with oligohydramnios were admitted to Imam Reza and Ummolbaninn hospitals. 196 patients were excluded despite the diagnosis of oligohydramnios due to refusal to participate in the investigation or exclusion criteria, and finally, 44 patients were enrolled.

The demographic information of the patients was recorded (age, BMI, gestational age). Then ultrasound sonography was performed to confirm the oligohydramnios diagnosis (AFI<5 cm). This procedure was conducted by one professor at Imam Reza Hospital and one at Ummol Banin Hospital.

Patients were randomized to intervention and control groups using a random number table. Then the mother's blood sample was collected to measure hematocrit, sodium, potassium, and urine samples to determine the specific gravity. In the intervention group, if the mother's urine specific gravity was equal to or less than 1010, 20 ml/kg of water was administered orally (as a loading dose for two hours) alongside with single inhalational dose of Desmopressin spray (Demex® brand produced by Sinadarou Labs Company, Tehran, Iran) that equals 10µg DDAVP sprayed in the nasal cavity and rubbed for one minute. The hematocrit, electrolyte levels, blood pressure and urine specific gravity, were measured 3h and 8h after baseline. If the specific gravity did not rise above 1010, another dose of Desmopressin was administered 12h after the first dose. The control group received intravenous maintenance fluid (5% dextrose, 35 cc/kg/day). The AFI was measured at baseline, 24 and 48 hours after study initiation. The specialist who conducted the sonography and the data analyst was unaware of the distribution of patients within groups.

### Statistical analysis

SPSS Statistics, version 16.0 (SPSS Inc., Chicago, Ill., USA) was used to analyze the data. Descriptive analysis included mean, standard deviation, frequency and percentage. Continuous variables were checked for normality before applying parametric (independent samples t-test) or non-parametric (Mann-Whitney U test) tests to compare quantitative variables between the two groups. Similarly, and considering the normality distribution, repeated ANOVA or Friedman test was used to compare the changes in outcomes at different points in time. In repeated measures of ANOVA, the Greenhouse-Geisser results were reported if the sphericity was not met in Mauchly's sphericity test. Otherwise, the sphericity assumed the test was reported. All tests were two-tailed, and a p-value below 0.05 was considered statistically significant.

## RESULTS

During this study, 240 patients diagnosed with oligohydramnios were admitted to Imam Reza and Ummolbaninn hospitals. 196 (81%) patients were excluded despite the diagnosis of oligohydramnios following exclusion criteria or because they did not consent to participate in the investigation. Finally, 44 (19%) patients were enrolled and divided into two equal groups. No significant difference was observed in the basic characteristics of the groups (Table 1).

**Table 1:** Comparison of basic characteristics in the two study groups.

	Intervention group (n=22)	Control group (n=22)	P-value
Age (years)	28.7±4.7	26.4±4.4	0.11*
Weight (kg)	68.3±8.3	69.8±9.3	0.66**
Height (cm)	160.6±5.7	160.8±5.4	0.89*
Body mass index (kg/m^2^)	26.5±3.4	27.0±3.6	0.53**
Gestational age (LMP)	231.4±16.4	226.9±17.7	0.39*
Gestational age (Sono)	230.4±16.7	226.5±17.4	0.46*

* – Independent samples t-test; ** – Mann-Whitney.

**Figure 1:**
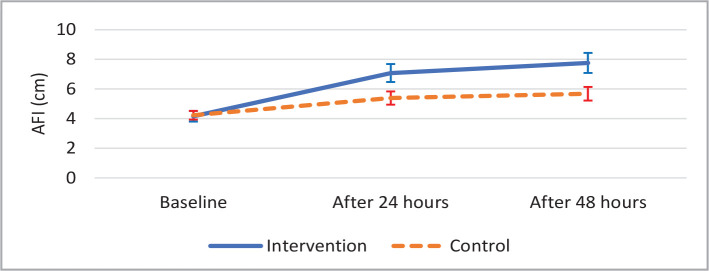
Trend of Amniotic Fluid Index (AFI) with CI95%.

The changes in AFI significantly increased in the intervention and control groups. However, as shown in Table 2, the increasing pattern was significantly different between the groups (Greenhouse-Geisser, p<0.001) (Figure 1).

**Table 2:** Changes in the amniotic fluid index (AFI) during the study in the two groups.

	Before treatment	24 hours after treatment	48 hours after treatment	P-value
Desmopressin	4.16±0.865	7.08±1.453	7.76±1.626	p<0.001 ^¥^
Control	4.23±0.702	5.39±1.079	5.68±1.108	p<0.001 ^¥^
P-value**	p<0.001 ^¥^			

Data are expressed as standard deviation±mean. * – Compare the pattern of change in each group separately; ** – Comparison of the pattern of changes between the two groups; ^¥^ – Using the Greenhouse-Geisser test.

Serum sodium levels significantly declined in the intervention (p<0.001) but not in the control group (p=0.07). Consequently, the between-group analysis for sodium showed a statistically different pattern (p<0.001). Potassium had a reduction pattern in both groups, but neither was statistically significant. Besides, the between-group analysis did not show a significant pattern (p=0.89). Hematocrit declined in the intervention (p<0.001) and control groups (p=0.02). Although the reduction rate was higher in the intervention group, no statistically significant difference was observed for the between-group pattern (p=0.23). Systolic blood pressure had an increasing pattern in the intervention (p<0.001) and control (p=0.005) groups. Nevertheless, no significant difference was found between-group for this pattern (p=0.21). On the other hand, diastolic blood pressure neither had a within-group difference nor between-group differences (p=0.97) (Table 3).

**Table 3:** Comparison of laboratory results between two groups.

		Baseline	After 3 hours	After 8 hours	P-value
Sodium (mEq/L)	Intervention	141.0±2.9	138.4±2.5	136.7±2.4	<0.001*
	Control	138.5±2.9	138.3±1.9	137.5±1.1	0.07*
Potassium (mEq/L)	Intervention	3.7±0.3	3.7±0.2	3.7±0.2	0.61*
	Control	3.9±0.2	3.9±0.4	3.8±0.2	0.49**
Hematocrit (%)	Intervention	36.5±2.1	34.8±2.0	33.9±2.1	<0.001**
	Control	36.5±2.0	35.5±1.6	35.0±1.9	0.02**
Systolic blood pressure (mmHg)	Intervention	106.4±9.0	109.1±9.3	112.5±10.0	<0.001**
	Control	103.6±7.2	105.9±7.6	107.3±8.6	0.005**
Diastolic blood pressure (mmHg)	Intervention	67.7±8.6	70.2±7.9	69.5±7.7	0.20**
	Control	66.4±7.8	68.6±7.2	68.2±6.9	0.18**

Comparisons are made with Repeated measures of ANOVA: * – Sphericity Assumed; ** – Greenhouse-Geisser.

**Figure 2:**
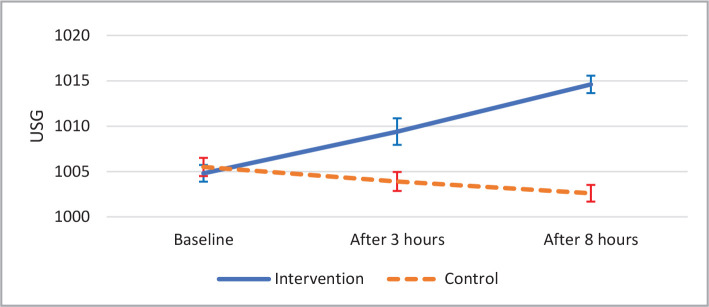
Trend of Urine Specific Gravity (USG) with CI95%.

As shown in Table 4, urine-specific gravity had an increasing pattern in the intervention group (p<0.001) and a decreasing pattern in control (p<0.001). The between-group difference was also significantly different (p<0.001) (Figure 2).

**Table 4:** Changes in the urine specific gravity in the two groups.

	Before treatment	3 hours after treatment	8 hours after treatment	P-value*
Desmopressin	1004.8±2.26	1009.4±3.57	1014.6±2.32	p<0.001 ^§^
Control	1005.5±2.44	1003.9±2.54	1002.6±2.28	p<0.001 ^§^
P-value**	p<0.001 ^§^			

Data are expressed as standard deviation±mean. * – Compare the pattern of changes in each group separately; ** – Comparison of the pattern of changes between the two groups; ^§^ – Using the Sphericity Assumed test.

## DISCUSSION

Oligohydramnios is an abnormally decreased amount of amniotic fluid that complicates 1 to 2 percent of pregnancies [2]. One of the most common threats to fetal health is oligohydramnios. Oligohydramnios is connected with intrauterine growth limitation, respiratory distress syndrome, post-maturity syndrome, and chronic fetal hypoxia [4]. Intravenous (iv) fluid loading was used as a means of volume expansion in women who were dehydrated or hypovolemic, and it corrected oligohydramnios and maternal plasma volume. Notably, amniotic fluid content correlates with maternal plasma volume and minimizes fetal complications [18].

In a study by Mohamed et al. (2021), oral hydration therapy improved oligohydramnios status in pregnant women. Moreover, this method also improved renal and uterine artery pulsatility indices [19].

This study aimed to determine the efficacy of inhaled Desmopressin in pregnant females with idiopathic oligohydramnios. Due to the effects of oligohydramnios on pregnancy and the effect of Desmopressin on maternal and fetal blood osmolarity and fetal urine output, this work investigated the therapeutic impact of nasal Desmopressin (DDAVP) in pregnant women with idiopathic oligohydramnios at 28 to 37 weeks of gestation.

A decrease in sodium plasma levels was observed in the Desmopressin group. Although plasma sodium levels were reduced in the treatment group with hydration, the changes were not statistically significant. A significant difference was observed in the changes in plasma sodium levels between Desmopressin and control groups. Nonetheless, the mean value of plasma sodium was still above 135 mEq/L (normal range) after the treatment in both groups.

The effect of Desmopressin was proved on plasma sodium depletion in animals and humans in previous studies [17, 20–22]. Hyponatremia production and plasma sodium level reduction in the context of Desmopressin prescription are probably related to the dosage and form of the medication. Hyponatremia seems to be due to the long-term high-dose administration of Desmopressin, and the therapeutic form of the medication is also more effective in developing hyponatremia.

However, Choi et al. (2015) showed that the administration of low-dose Desmopressin could also cause hyponatremia [23]. Moreover, Kataoka et al. (2014) stated that hyponatremia incidence is higher in the forms of injection and oral administration. Nonetheless, Desmopressin inhalation may also decrease the plasma sodium level and increase hyponatremia incidence [24].

Desmopressin can increase the water reabsorption of urinary tract tubes in the kidney, potentially affecting the vasopressin receptors 2 (through agonistic effects), which leads to a decrease in sodium plasma levels [25]. On the other hand, animal studies showed that Desmopressin, with an effect on the sodium-chloride pump, increases sodium reabsorption from the nephron, leading to an increase in the serum sodium level and *vice versa*.

Ross et al. investigated the Desmopressin impact on plasma sodium levels in mothers with oligohydramnios. In this study, an asymptomatic decrease in plasma sodium was seen as a result of Desmopressin treatment [21]. The results of the two studies confirmed the finding above [26, 27]. There seem to be other mechanisms for investigating the effect of this drug on plasma sodium levels, some of which are mentioned in previous studies. However, there has been a debate about their precision.

In the current work, the changes in plasma potassium were not significant in any of the studied groups. Furthermore, no between-group difference was observed. However, in the Desmopressin and control groups, the potassium level decreased compared to baseline. Previous studies revealed that Desmopressin, in addition to sodium and chlorine, can also affect the plasma levels of some other ions, including potassium [27]. It has been suggested that Desmopressin can influence potassium secretion in the distal nephron segment. Nonetheless, the precise mechanism of this effect and its effective vasodilators are still being investigated.

In a study by Kutina et al. (2013), Desmopressin could increase the urinary excretion of potassium [28]. However, due to the accurate adjustment of potassium in the body, its increase in urine does not significantly affect plasma potassium levels, which was also observed in the current research.

Moreover, the AFI level in the Desmopressin group increased after 24 and 48h of treatment. The mean value of AFI in the control group increased after 24 and 48 h. The increase rate in AFI was, on the other hand, higher in the Desmopressin group after 24 and 48 h than before treatment. This issue has also been investigated in other studies. For example, Ross et al. (1996) conducted a study in the US to investigate the Desmopressin impact on oligohydramnios in pregnant women. Their study revealed that oral fluids and Desmopressin could reduce the maternal plasma osmolality and significantly increase amniotic fluid volume and can be used to treat oligohydramnios [15]. Other animal studies confirmed this finding [15, 26, 27], indicating that Desmopressin is superior to the serum in treating oligohydramnios.

However, a thorough review of the effect mechanism and the potential side effects of this medicine must be carefully identified before clinical use. In previous studies, Desmopressin did not cross the placenta of sheep, and the probability that the drug can pass through the placenta in humans is, therefore, very low. So, this drug will not directly affect the fetus [26].

The antidiuretic effect of Desmopressin causes water retention in a mother's body that induces the maternal plasma hypo-osmolality. Given that the embryo and the mother are linked and change together, this decrease will also occur in the osmolality of the embryo body. On the other hand, since Desmopressin does not have antidiuretic effects on the fetus, reducing the osmolality of the fetal plasma causes urination, which ultimately leads to increased urine output in the fetus and the treatment of oligohydramnios [16]. It can be suggested that the effects of intravenous Desmopressin should also be studied. According to Abbasalizadeh et al. (2015), there was no significant change in the mean AFI between the two groups (hydration and Desmopressin) at baseline, 48 hours later, one week later, and two weeks later. A substantial increase in AFI was seen in both groups during therapy [29]. This was not consistent with our study because only one or two doses of Desmopressin were applied in the present research, and patients were evaluated only during the treatment period.

One of the limitations of this study was that it only evaluated one or two doses of DDAVP; other doses can be evaluated in future projects. Also, studying maternal and fetal outcomes like delivery type, neonatal weight, APGAR, and admission to NICU can be of great interest.

## CONCLUSION

This study showed that 10 µg Desmopressin inhalation could increase the amniotic fluid and enhance the treatment of oligohydramnios in pregnant women, compared to the serum administration.

## Personal thanks

This study was supported by the Deputy of Research of the Mashhad University of Medical Sciences. The authors appreciate the help of nurses at Imam Reza and Ummol Banin Hospitals. We also appreciate the statistical and methodologic support from the Clinical Research Development Unit of Akar Hospital for revising this manuscript.
